# In Vitro Neuroprotective Effects of a Mixed Extract of Bilberry, *Centella asiatica*, *Hericium erinaceus*, and Palmitoylethanolamide

**DOI:** 10.3390/foods14152678

**Published:** 2025-07-30

**Authors:** Rebecca Galla, Sara Ferrari, Ivana Miletto, Simone Mulè, Francesca Uberti

**Affiliations:** 1 Noivita Srls, Spin Off of University of Piemonte Orientale, Strada Curti 7, 28100 Novara, Italy; 2 Laboratory of Physiology, Department for Sustainable Development and Ecological Transition, University of Piemonte Orientale, UPO, 13100 Vercelli, Italy; 3 Department of Pharmaceutical Sciences, University of Piemonte Orientale, Largo Guido Donegani 2/3, 28100 Novara, Italy

**Keywords:** natural extracts, oral supplementation, anti-inflammatory, neurotrophins, oxidative stress

## Abstract

Oxidative stress, driven by impaired antioxidant defence systems, is a major contributor to cognitive decline and neurodegenerative processes in brain ageing. This study investigates the neuroprotective effects of a natural compound mixture—composed of *Hericium erinaceus*, Palmitoylethanolamide, Bilberry extract, and *Centella asiatica*—using a multi-step in vitro strategy. An initial evaluation in a 3D intestinal epithelial model demonstrated that the formulation preserves barrier integrity and may be bioaccessible, as evidenced by transepithelial electrical resistance (TEER) and the expression of tight junctions. Subsequent analysis in an integrated gut–brain axis model under oxidative stress conditions revealed that the formulation significantly reduces inflammatory markers (NF-κB, TNF-α, IL-1β, and IL-6; about 1.5-fold vs. H_2_O_2_), reactive oxygen species (about 2-fold vs. H_2_O_2_), and nitric oxide levels (about 1.2-fold vs. H_2_O_2_). Additionally, it enhances mitochondrial activity while also improving antioxidant responses. In a co-culture of neuronal and astrocytic cells, the combination upregulates neurotrophic factors such as BDNF and NGF (about 2.3-fold and 1.9-fold vs. H_2_O_2_). Crucially, the formulation also modulates key biomarkers associated with cognitive decline, reducing APP and phosphorylated tau levels (about 98% and 1.6-fold vs. H_2_O_2_) while increasing Sirtuin 1 and Nrf2 expression (about 3.6-fold and 3-fold vs. H_2_O_2_). These findings suggest that this nutraceutical combination may support the cellular pathways involved in neuronal resilience and healthy brain ageing, offering potential as a functional food ingredient or dietary supplement.

## 1. Introduction

Age-related cognitive decline is a condition that varies in different cognitive domains and has considerable disparities among the elderly [1]. Epigenetic changes are influenced by biological, environmental, and lifestyle factors, leading to phenotypic variations as individuals age and an increase in cognitive decline and neurodegenerative diseases [2,3]. Ageing-related cognitive impairment is further exacerbated by changes in astrocyte activity and a decline in brain-derived neurotrophic factor (BDNF). BDNF-mediated signalling has been the subject of extensive research due to its significance in neurogenesis, synaptic plasticity, and learning and memory processes, all of which are associated with age-related cognitive decline. The primary effects induced by BDNF are mediated by the tropomyosin receptor kinase (Trk) family receptor [4,5,6]. Modifications in nerve growth factor (NGF) signalling can also be due to ageing, affecting the survival and proliferation of cholinergic neurons in the basal forebrain [7].

Further, it has been extensively shown that there is a direct correlation between an increase in free radicals and oxidative stress and the development of neurodegenerative diseases [8]. Indeed, according to Harman’s free radical hypothesis of ageing, oxidative damage caused by reactive oxygen species (ROS) to cellular macromolecules such as DNA, proteins, and lipids results in ageing-related declines in biochemical and physiological function [9]. As the brain ages, a redox imbalance resulting from increased ROS production and decreased antioxidants leads to age-related diseases. ROS can impair cellular activity by altering proteins and lipids in the brain; indeed, ROS-induced lipid peroxidation occurs in the brains of elderly humans and animals with central nervous system (CNS) dysfunction, such as cognitive impairment [10]. Organisms can age more quickly if their antioxidant defences, such as superoxide dismutase (SOD) and glutathione peroxidase (GPx), are compromised and they cannot eliminate oxidatively damaged macromolecules [10].

The brain is susceptible to oxidative stress [11,12] due to its high oxygen consumption, rapid glucose metabolism, and elevated levels of redox-active iron in specific regions [13]. The antioxidant activity of several natural extracts to reduce free radical levels and metal binding is thus critical [14]. A growing body of scientific evidence suggests that several natural extracts, because of their bioactive components, can also activate specific compensatory mechanisms to halt brain degeneration [15,16,17,18]. These active components interact with molecular targets during brain cell ageing, restoring neurotrophin production (BDNF, NGF and Neurotrophin-3) and tissue function [19,20]. These components influence neurotrophin expressions and growth factors, supporting neuronal activity and brain function [21].

Recent research suggests dietary treatments may prevent or reduce age-related neurological deterioration [11]. Mushrooms have been eaten and used medicinally for millennia [21,22]. They thus serve as a valuable source of physiologically active substances that function as nutraceuticals and food. Phenolic compounds and polysaccharides provide edible mushrooms with antioxidant capabilities. Among the mushrooms known for medicinal benefits, including antioxidant effects, is *Hericium erinaceus* [23]. The primary active components of the body and mycelium of *Hericium erinaceus* include polysaccharides, erinacins, hericerins, steroids, alkaloids, and lactones; in particular, erinacines A-I, mainly in tisoform C, stimulate the production of NGF and BDNF in astrocytes, leading to a range of physiological effects [24,25]. In vitro and preclinical research have shown that *Hericium erinaceus* possesses neuroprotective properties in neurodegenerative disorders. Furthermore, some bioactive compounds present in *Hericium erinaceus*, such as Erinacins and Hericenones, cross the blood–brain barrier (BBB) [26,27], thereby increasing the synthesis of trophic factors such as NGF and BDNF [21].

Palmitoylethanolamide (PEA) is another chemical that reduces cognitive deterioration at the nervous level. PEA is a biosynthesised compound [28] that helps maintain cellular homeostasis and improves cognitive functions, such as memory and learning, by reducing oxidative stress and pro-inflammatory markers, rebalancing glutamatergic transmission, and modulating lipid metabolism [29,30].

Age-related cognitive health is also supported by some traditional botanical treatments, including *Centella asiatica* [31]. This plant has been extensively researched for its potential active compounds (such as asiaticocide and asiatic acid [32]) in various fields, including neuroprotective [33], antinociceptive [34], antioxidant, antihyperlipidemic [35] and anticancer activities [36]. *Centella asiatica* has been shown to have neuroprotective properties [37], making it a promising treatment for cognitive decline and other nervous system deterioration [38]. It induces BDNF production, alleviating memory impairment associated with cognitive decline and promoting antioxidant genes for cognitive enhancement [39].

Since the leading causes of brain ageing are determined by an increase in oxidative stress, some studies explore the opportunity to use strong antioxidant extracts to restore brain homeostasis [40,41]. In this context, the potential role of Bilberry (*Vaccinium myrtillus* or *V. myrtillus*, commonly known as Blueberry) has been highlighted due to its high content of anthocyanins [42,43]. As the literature indicates, Bilberry is a highly concentrated source of phenolic acids, flavonoids, phenolic glycosides, triterpenoids, carotenoids, organic acids, carbohydrates, and higher fatty acids. Phenolic compounds and phytocompositions based on total phenols are increasingly employed in neurological therapy to treat and prevent neurodegenerative disorders and cerebral ischemia [44]. Human studies have revealed beneficial behavioural outcomes: after 12 weeks of Bilberry juice supplementation, older individuals with mild cognitive impairment demonstrated improved memory and learning capabilities compared to the placebo group [45]. This can be explained by its ability to modulate gene expression in memory processes, such as calmodulin-dependent kinase II [46].

The concept of the gut–brain axis, characterised as a complex interactive network between the gastrointestinal system and the central nervous system, is gaining recognition in the in vitro research concerning cognitive decline and neurodegenerative diseases [47]. Investigations conducted on murine models indicate that heightened permeability of the intestinal barrier, resulting from ageing, permits the translocation of amyloids produced by bacteria into the bloodstream, thereby exacerbating brain inflammation and impairing cognitive function [48,49]. Moreover, findings from meta-analyses suggest that intestinal metabolites may contribute to inflammation and the aggregation of tau and amyloid proteins in neurodegenerative brain disorders [50].

Therefore, this study aims to evaluate the neuroprotective effects of a mixture (MIX) composed of *Hericium erinaceus*, PEA, Bilberry extract, and *Centella asiatica* using a multi-step in vitro approach, with the ultimate goal of exploring its potential to counteract oxidative stress and inflammation associated with brain ageing, and to promote healthy cognitive function.

## 2. Materials and Methods

### 2.1. Agent Preparations

*Hericium erinaceus*, PEA (simbiΩ), Bilberry extract and *Centella asiatica* (all donated by Natural Bradel S.r.l.) were tested individually and in combination to create a new formulation to reach the brain after crossing the intestinal barrier (for more details see Appendix A). *Hericium erinaceus* was tested ranging from 100 µg/mL to 400 µg/mL [51], PEA ranging from 0.1 µM to 0.4 µM [52], Bilberry extract, ranging from 100 µg/mL to 500 µg/mL [53], and *Centella asiatica* extract ranging from 250 µg/mL to 1000 µg/mL [54]. All the substances were prepared directly in Dulbecco’s Modified Eagle’s Medium (DMEM, Merck Life Science, Rome, Italy) without phenol red and supplemented with 0.5% foetal bovine serum (FBS; Merck Life Science, Rome, Italy), 2 mM L-glutamine (Merck Life Science, Rome, Italy), and 1% penicillin–streptomycin (Merck Life Science, Rome, Italy) for all analyses. All the substances were prepared at 10× concentration and then diluted to the appropriate concentration. They were prepared ready-to-use and re-prepared for each independent experiment. Following the identification of the most effective concentration for each tested substance, the selected concentrations were subsequently employed both individually and in combination. The combined formulation, hereafter referred to as “Mix”, consisted of 400 μg/mL *Hericium erinaceus*, 100 μg/mL bilberry extract, 250 μg/mL *Centella asiatica*, and 0.2 μM PEA.

### 2.2. Cell Cultures

The human intestinal epithelial barrier is often modelled using the human colorectal cancer cell line Caco-2 (ATCC, Manassas, VA, USA) [55,56,57]. This cell line was chosen because the Caco-2 cell line is a widely recognised in vitro model for assessing drug permeability across the small intestinal epithelium. Caco-2 cells spontaneously differentiate into enterocyte-like cells morphologically and demonstrate a substantial correlation between human jejunal absorption and Caco-2 cell permeability for passively absorbed pharmaceuticals [58]. Caco-2 cells were cultured in Adv DMEM-F12 (Advanced Dulbecco’s Modified Eagle’s Medium/Nutrient F-12 Ham, GIBCO^®^ ThermoFisher Scientific, Waltham, MA, USA) containing 10% FBS, 2 mM L-glutamine, and 1% penicillin–streptomycin in an incubator at 37 °C and 5% CO_2_. To maintain the integrative qualities of paracellular permeability and transport, comparable to the intestinal absorption mechanism following oral intake in humans, the cells utilised for the tests had a passage number between 26 and 32 [59]. A 96-well plate with 1 × 10^4^ cells was used to assess cell viability using an MTT-based In Vitro Toxicology Assay Kit (Merck Life Science, Rome, Italy). To synchronise the cells, they were cultured in DMEM without red phenol and 0.5% FBS (GIBCO^®^ ThermoFisher Scientific, Waltham, MA, USA), 2 mM L-glutamine, and 1% penicillin–streptomycin (Merck Life Science, Rome, Italy) at 37 °C eight hours before stimulation. To conduct absorption analyses, 2 × 10^4^ cells were plated on a 6.5 mm Transwell^®^ (Corning^®^ Costar^®^, Merck Life Science, Rome, Italy) with 0.4 μm pore polycarbonate membrane inserts in a 24-well plate [60]. The EMA and FDA have approved this in vitro model for predicting human substances’ absorption, metabolism, and bioavailability following oral administration [60,61,62].

SH-SY5Y cells (ATCC, Manassas, VA, USA) are derived from human neuroblastoma cells. They are widely used in vitro model systems for studies on neurophysiology, testing of pharmacological preparations, and investigating the effects of toxins on neuronal viability and their relation to neurodegenerative diseases [63,64]. This is because they are a simple and inexpensive model for biochemical and cellular investigations of ageing in vitro [63]. SH-SY5Y cells were cultured in a 1:1 mixture of Advanced Dulbecco’s Modified Eagle Medium F12 (Adv DMEM F12; GIBCO^®^ ThermoFisher Scientific, Waltham, MA, USA) and Adv DMEM, supplemented with 10% FBS, 2 mM HEPES, and 2 mM The cells were incubated at 37 °C with 5% CO_2_ and 95% humidity [65]. Passages 3 and 20 contained the cells employed in these investigations. The cells were plated differently to conduct multiple experiments in the gut–brain axis assay. Precisely, 1 × 10^4^ cells were placed in 96-well plates to investigate cell viability using an MTT-based In Vitro Toxicology Assay Kit (Merck Life Science, Rome, Italy), Reactive Oxygen Species (ROS) production using cytochrome C (Merck Life Science, Rome, Italy) in a complete medium. NO production using a colourimetric Assay kit. Additionally, using an ELISA kit, 1 × 10^5^ SH-SY5Y cells/well were plated in the 24-well plates to study inflammation and lipid peroxidation. The cells were synchronised in DMEM (GIBCO^®^ ThermoFisher Scientific, Waltham, MA, USA) without red phenol and supplemented with 0.5% FBS, 2 mM L-glutamine, and 1% penicillin–streptomycin at 37 °C eight hours before stimulation. To determine if the mix and single agents could counteract the damage induced by oxidative stress conditions, the cells were treated with 200 µM H_2_O_2_ for 30 min before stimulation [66].

The human astrocyte cell line CCF-STTG1 purchased from American Type Culture Collection (ATCC, Manassas, VA, USA) derived from the brain of a 68-year-old astrocytoma patient and were cultured in flasks in Roswell Park Memorial Institute medium (RPMI, Merck Life Science, Rome, Italy) supplemented with 10% of FBS (Merck Life Science, Milan, Italy), supplemented with 2 mM Hepes (Merck Life Science, Rome, Italy), 2 mM L-Glutamine (Merck Life Science, Rome, Italy) and 1% P/S (Merck Life Science, Rome, Italy). These cells were co-cultured with the SH-SY5Y cell line to study specific intracellular pathways correlated with cognitive decline.

### 2.3. Experimental Protocol

**Figure 1:**
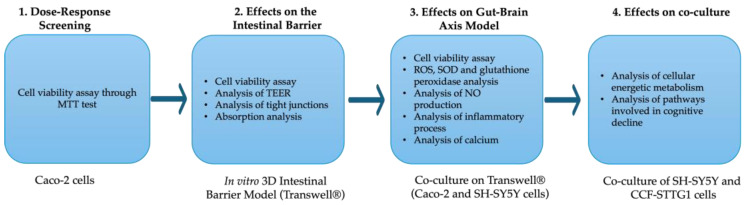
A schematic representation of the experimental methodology is divided into phases.

The study was divided into four phases (Figure 1). This study investigated the biological activities of PEA, *Hericium erinaceus*, *Centella asiatica*, and Bilberry extract in mitigating cognitive decline and neurodegeneration mechanisms after oral intake. To accomplish this, the substances were evaluated either individually or in combination. Considering that the goal of this study is to assess the possibility of developing a new nutraceutical formulation, the first step employed CaCo-2 cells as intestinal cells that were used to mimic intestinal absorption. The first experiment was a time and dose-dependent study on the cell viability of CaCo-2 cells with *Hericium erinaceus* (ranging from 100 µg/mL to 400 µg/mL [51]), Bilberry extract (ranging from 100 µg/mL to 500 µg/mL [53]), *Centella asiatica* extract (ranging from 250 µg/mL to 1000 µg/mL [54] and PEA (ranging from 0.1 µM to 0.4 µM [52]) to determine the appropriate concentration of these compounds following stimulations; these analyses were performed in a timeframe from 1 h to 6 h. During the analyses, the cells were maintained in DMEM without red phenol and 0.5% FBS (GIBCO^®^ ThermoFisher Scientific, Waltham, MA, USA), 2mM L-glutamine, and 1% penicillin–streptomycin (Merck Life Science, Rome, Italy). Then, the beneficial concentrations of the agents were tested on the intestinal barrier model alone and in combination to analyse cell viability, the integrity of the epithelial barrier by Trans-Epithelial Electrical Resistance (TEER), and specific tight junctions (TJs), demonstrating that these extracts do not cause damage to the intestinal barrier. Then, we investigate the permeability through the intestinal barrier (from 1 h to 6 h) to demonstrate that these extracts and their combinations are correctly absorbed and distributed. In a second set of experiments, the biological activity and beneficial effects of the new formulation were evaluated after intestinal absorption, directly on the gut–brain axis, to assess its capacity to counteract mechanisms linked to cognitive decline and neurodegeneration [57]. To mimic the in vivo condition, the neuronal cells were pretreated with 200 µM H_2_O_2_ for 30 min [66]. Precisely, the ability to restore the damages caused by oxidative stress of PEA, *Hericium erinaceus*, *Centella asiatica*, and Bilberry extract, alone and in combination, was investigated through cell viability analysis, SOD levels and ROS, GPx and NO production analysis. Furthermore, lipid peroxidation and inflammatory biomarkers were analysed, as there is a correlation between oxidative stress and increased inflammatory cytokines and calcium levels, as well as cognitive disorders. All the analyses were performed at 24 h. During the analyses, the cells were maintained in DMEM without red phenol and 0.5% FBS (GIBCO^®^ ThermoFisher Scientific, Waltham, MA, USA), 2 mM L-glutamine, and 1% penicillin–streptomycin (Merck Life Science, Rome, Italy).

In addition, a co-culture of SH-SY5Y and CCF-STTG1 was analysed for cellular energy metabolism, as impaired or inefficient energy metabolism can impair cognitive abilities. BDNF and NGF production, as well as the activation and neurodegenerative markers, were examined, as neurotrophins have a range of effects on neuronal function. All analyses were performed at 24 h. During the analyses, the cells were maintained in DMEM without red phenol and 0.5% FBS (GIBCO^®^ ThermoFisher Scientific, Waltham, MA, USA), 2 mM L-glutamine, and 1% penicillin–streptomycin (Merck Life Science, Rome, Italy). To mimic the in vivo condition, the cells were pretreated with 200 µM H_2_O_2_ for 30 min.

### 2.4. MTT Cell Viability Assay

The literature [67] stated that the MTT test was used to assess cell viability after stimulation. Cells were cultured in an incubator in DMEM without phenol red, 0% FBS, and 1% MTT probe for 2 h at 37 °C. Cell viability was measured using a spectrometer (Infinite 200 Pro-MPlex, Tecan, Männedorf, Switzerland) by measuring absorbance at 570 nm and 690 nm. Results were presented as mean ± SD (%) compared to control (line 0, untreated cells) from five independent triplicate experiments.

### 2.5. ROS Production

The ROS generated following stimulations were examined using the rate of superoxide anion release [67]. Absorbance was determined at 550 nm using a spectrometer (Infinite 200 Pro-MPlex, Tecan, Männedorf, Switzerland). Results are presented as mean ± SD (%) compared to control (line 0, untreated cells) from five independent triplicate tests.

### 2.6. Integrity Analysis on an Intestinal Barrier In Vitro Model

Caco-2 cells were used to create an intestinal barrier model to examine how test compounds crossed the barrier. EVOM3 with STX2 rod electrodes (World Precision Instruments, Sarasota, FL, USA) was used to measure TEER values. The assay was conducted every two days for 21 days until a TEER value ≥ 400 Ω·cm^2^ was obtained before stimulation [68]. This is necessary for the exposure of intestinal villi, cell differentiation, and monolayer formation. On day 21, the apical and basolateral culture media were switched to produce distinct pH levels: approximately 6.5 at the apical level, which mimics the small intestine’s lumen (an acidic pH), and approximately 7.4 at the basolateral level, which mimics human blood (a neutral pH) [69]. Before the experiment began, the TEER values were tested again to confirm that the readings had stabilised after the cells had been maintained at 37 °C and 5% CO_2_ for 15 min. At the end of the stimulation, the basolateral medium was collected and used to stimulate the central nervous system model. In detail, TEER measurement is used to assess the barrier function of epithelial cells on these porous media. This test was performed every 2 days for 21 days until a TEER value ≥ 400 Ω·cm^2^ was reached before stimulation. A Transwell with the same growth medium as the cells but without cells was used to eliminate background noise caused by the semi-porous membrane, allowing for the accurate determination of TEER values.

When the threshold value was reached, the fluorescent probe, 0.04% fluorescein (Merck Life Science, Rome, Italy) [70], was added along with the treatments. At excitation/emission wavelengths of 490/514 nm, fluorescence at the basolateral level was detected using a fluorescence spectrophotometer (Infinite 200 Pro-MPlex, Tecan, Männedorf, Switzerland). The outcomes are given as a percentage of the initial amount that entered the cells. Using the following formula, the permeation rate [nmol min (mg protein)], J, was determined using a methodology that has been validated in the literature [71]:

J = (Jmax × [C])/(Kt + [C])

where

C—initial fluorescein concentration.

Jmax—maximum permeation rate.

Kt—Michaelis–Menten constant.

The results are then expressed as mean ± SD (%) compared to the control sample (untreated cells).

Negative controls without cells were analysed to exclude the influence of Transwell membranes.

### 2.7. Human Tight Junction Analysis

Following the manufacturer’s instructions, the Caco-2 lysates were subjected to occludin activity analysis using the Human Occludin (OCLN) ELISA Kit (MyBiosource, San Diego, CA, USA), claudin-1 level analysis using the Cusabio Technology LCC ELISA Kit, Huston, Houston, TX, USA, and Zona Occludens 1 (ZO-1) activity analysis using the human tight junction protein 1 (TJP1) ELISA kit (MyBiosource, San Diego, CA, USA). A spectrophotometer (Infinite 200 Pro-MPlex, Tecan, Männedorf, Switzerland) was used to measure the absorbance at 450 nm. The data were compared to the standard curve, which ranges from 0 to 1000 pg/mL for ZO-1 and claudin-1 and from 0 to 1500 pg/mL for occludin. The results were presented as percentages (%) compared to the control (0 line, untreated cells) in five independent experiments conducted in triplicate [59].

### 2.8. Gut–Brain Axis Model

Using a typical methodology outlined in the literature, the Caco-2 and SH-SY5Y cell lines were co-cultured on a Transwell^®^ device to construct the gut–brain axis [72]. In particular, the Transwell system was utilised to divide the two chambers containing ADV DMEM media (Merck Life Sciences, Rome, Italy) using a semipermeable membrane with a 0.4 μm pore size (Corning Costar, Merck Life Science, Rome, Italy). 2.5 × 10^4^ Caco-2 cells were plated on the insert: the cells used were between 26 and 32 passages and were maintained in Adv DMEM-F12 (GIBCO^®^ ThermoFisher Scientific, Waltham, MA, USA) containing 10% FBS, 2 mM L-glutamine, and 1% penicillin–streptomycin in an incubator at 37 °C and 5% CO_2_. Instead, 4 × 10^2^ SH-SY5Y cells/well were plated in an independent 24-well on the seventh development day: the cells used from the experiments were between 33 and 20 passages and were maintained in a mix 1:1 of Adv DMEM F12 (GIBCO^®^ ThermoFisher Scientific, Waltham, MA, USA) and Adv DMEM, supplemented with 10% FBS, 2 mM HEPES, and 2 mM and incubated at 37 °C with 5% CO_2_ and 95% humidity. After 14 days, the intestinal epithelium has matured, acquiring a high TEER value (≥400 Ω·cm^2^), indicating the development of tight junctions. Both cell lines were cultivated independently for 5 days before being co-incubated for 15 h. TEER was re-evaluated when the two lines were joined before stimulation as a precaution, as opposed to any modification of the intestinal cell monolayer. Subsequently, the cells were subjected to different experiments.

### 2.9. Analysis of Nitric Oxide Production

To quantify nitric oxide (NO) production, a Griess assay kit (Promega, Milan, Italy) was used to assess its production in SH-SY5Y supernatant after stimulation, following the manufacturer’s instructions [73]. Samples were analysed after pretreatment with 200 μM H_2_O_2_ to mimic oxidative stress associated with cognitive decline. The samples’ absorbance was measured using a spectrometer (Infinite 200 Pro-MPlex, Tecan, Männedorf, Switzerland) at 520 nm to 550 nm. The results were expressed as a percentage (%) normalised to the untreated samples based on the standard curve obtained with standard nitrate. The results were presented as the mean ± SD (%) versus the control (line 0, untreated cells) in five independent experiments performed in triplicate.

### 2.10. Lipid Peroxidation Analysis

The thiobarbituric acid reactive substance test (TBARS, Cayman Chemical Company, Ann Arbor, MI, USA) examined lipid peroxidation in SH-SY5Y cells [74]. The procedure involved adding 100 μL of the sample or standard to a 5 mL vial that contained 100 μL of SDS solution. After adding 4 mL of Dye Reagent to each vial, the vials were boiled for an hour and then allowed to sit on ice for 10 min. Following a 10 min centrifugation at 1600× *g* at 4 °C, 150 μL was added to each well of the 96-well plate, and the absorbance was measured at 530–540 nm using an Infinite 200 Pro-MPlex (Tecan, Männedorf, Switzerland). The findings were reported as a percentage (%) of the control (line 0, untreated cells) of five independent triplicate tests.

### 2.11. SOD Assay

Using SH-SY5Y lysate, the SOD level was determined following the manufacturer’s instructions (Cayman’s Superoxide Dismutase Assay Kit; Tallinn, Estonia) [75]. A standard curve (0.05–0.005 U/mL) was used to compare the data and determine the amount of SOD in the cell lysates. A spectrometer (Infinite 200 Pro MPlex, Tecan, Männedorf, Switzerland) was used to measure the absorbance of each sample at 440–460 nm. The data were presented as a percentage (%) of the control (line 0, untreated cells) of five independent triplicate tests.

### 2.12. Glutathione Peroxidase Assay

The level of GPX was measured on SH-SY5Y cell lysate following the manufacturer’s instructions (Cayman’s Superoxide Dismutase Assay Kit; Tallinn, Estonia) [60]. The absorbance of all samples was measured through a spectrometer (Infinite 200 Pro MPlex, Tecan, Männedorf, Switzerland) at 340 nm. The data were represented as a percentage (%) of the control (line 0, untreated cells) of five independent triplicate tests.

### 2.13. TNFα ELISA Kit

The TNFα concentration in SH-SY5Y cells was determined using the TNFα ELISA kit (Merck Life Science, Rome, Italy) according to the standard protocol [76]. Colourimetric intensity was measured at 450 nm using a spectrophotometer (Infinite 200 Pro-MPlex, Tecan, Männedorf, Switzerland). A calibration curve (24.58 pg/mL to 6000 pg/mL) was used to determine the results as a percentage relative to the control (line 0, untreated cells) in five independent triplicate tests.

### 2.14. NFkB ELISA Kit

The NF-kB (p65) Transcriptional factor Assay kit (Cayman Chemical Company, Ann Arbor, MI, USA) was used on SH-SY5Y lysates, following the manufacturer’s instructions [71]. The absorbance was measured using a spectrophotometer (Infinite 200 Pro-MPlex, Tecan, Männedorf, Switzerland) at 450 nm. The results were compared to the standard curve produced by the NF-kB transcription factor positive control (p65) (0 to 10 µL/well according to different scalar dilutions). The results of five independent tests in triplicate were presented as mean ± SD (%) compared to the control (line 0, untreated cells).

### 2.15. IL-1β ELISA Kit

SH-SY5Y cells were assessed using the IL-1β ELISA kit (R&D Systems, MN, USA) following the manufacturer’s instructions [77]. Using a plate reader (Infinite 200 Pro MPlex, Tecan, Männedorf, Switzerland), set to read the plate at 450 nm with correction at 570 nm, the samples’ optical density was compared to a standard curve (ranging from 3.906 to 250 pg/mL). The results of five independent tests, performed in triplicate, were presented as the mean ± SD (%) compared to the control (line 0, untreated cells).

### 2.16. IL-6 ELISA Kit

An IL-6 ELISA kit (eBioscience, San Diego, CA, USA) was used to measure the amount of IL-6 in the supernatant following the manufacturer’s instructions [78]. Tecan, Männedorf, Switzerland’s Infinite 200 Pro MPlex spectrometer recorded the greatest absorption wavelength at 450 nm. Results were displayed as a percentage (%) relative to the control (line 0, untreated cells), and concentration was represented as pg/mL on a standard curve (0.078 to 5 pg/mL).

### 2.17. Calcium Levels Assay

Analysis of calcium levels was performed according to the instructions provided in the Calcium Assay Kit (abcam, Cambridge, UK) [79]. The absorbance of the samples was measured at 575 nm using a microplate reader (Tecan, Männedorf, Switzerland’s Infinite 200 Pro MPlex). Results were displayed as a percentage (%) relative to the control (line 0, untreated cells).

### 2.18. Co-Culture CCF-STTG1/SH-SY5Y

The co-culture system of SH-SY5Y cells and CCF-STTG1 cells was established based on a previously published protocol [80]. Initially, astrocytes were plated at a density of 6 × 10^3^ cells per well onto 12-well plates precoated with Matrigel, using DMEM supplemented with 10% FBS and N-2. The cells were incubated overnight at 37 °C to allow for adherence. The next day, the medium was replaced, and SH-SY5Y cells were added at a concentration of 3 × 10^4^ cells per well. The co-cultures were then subjected to a 5-day differentiation period in medium supplemented with 10 µM retinoic acid (RA) and 1% FBS. To mimic cognitive decline conditions, the cells were pretreated with 200 µM H_2_O_2_ for 30 min.

### 2.19. Oxygen Consumption and Mitochondrial Membrane Potential

The oxygen consumption and mitochondrial membrane potential were immediately and simultaneously quantified by Oxygen Consumption/Mito membrane Potential Dual Assay Kit (Cayman Chemical Company, Ann Arbour, MI, USA) [66]. Oxygen consumption fluorescence was measured using excitation and emission at 380 nm and 650 nm, respectively. The membrane potential was measured using JC-1 red aggregates at an excitation/emission of 560/590 nm and monomers at an excitation/emission of 485/535 nm in a fluorescence spectrometer (Infinite 200 Pro MPlex, Tecan, Männedorf, Switzerland) The results of five independent tests in triplicate were presented as mean ± SD (%) compared to control (line 0, untreated cells).

### 2.20. ATP Assay

Following the manufacturer’s recommendations, the cells were treated right away with the ATP assay kit’s components (Calbiochem, San Diego, CA, USA) after each stimulation [76]. A spectrometer (Infinite 200 Pro MPlex, Tecan, Männedorf, Switzerland) was used to quantify luminescence one minute after the ATP monitoring enzyme was added. The luminescence was estimated as μmol of ATP/g protein40 and reported as means ± SD of nanomol (nmol) per well of five independent tests in triplicate.

### 2.21. BDNF ELISA Kit

BDNF quantification was performed in SH-SY5Y cells using the BDNF ELISA Kit (Thermo ScientificTM, Waltham, MA, USA), following the manufacturer’s instructions [81]. Using a spectrometer (Infinite 200 Pro-MPlex, Tecan, Männedorf, Switzerland) to measure absorbance at 450 nm, the quantity of BDNF was calculated by comparing the results with the BDNF standard curve (range 0.066–16 ng/mL). The results of five separate experiments conducted in triplicate were shown as a percentage (%) of the control (line 0, untreated cells).

### 2.22. NGF ELISA Kit

The quantification of NGF was measured in SH-SY5Y cells using an NGF ELISA Kit (Thermo ScientificTM, Waltham, MA, USA), following the manufacturer’s instructions [82]. A spectrometer (Infinite 200 Pro-MPlex, Tecan, Männedorf, Switzerland) was used to measure the absorbance at 450 nm to quantify the NGF concentration. The results were then compared to the standard curve (6.86–5000 pg/mL). Five separate tests were conducted in triplicate, and the results were shown as a percentage (%) compared to the control (line 0, untreated cells).

### 2.23. Amyloid Precursor Protein (APP) ELISA Kit

Quantification of amyloid precursor protein (APP) was measured in SH-SY5Y using the Amyloid Beta A4 protein ELISA kit (Merck, Milan, Italy) on cell supernatants, as reported in the literature [66]. APP concentration was evaluated by measuring absorbance at 450 nm with a spectrometer (Infinite 200 Pro-MPlex, Tecan, Männedorf, Switzerland). The APP standard curve was used to compare the data and determine the concentration. Results were expressed as a percentage (%) relative to the control (line 0, untreated cells) from five separate triplicate experiments.

### 2.24. Sirt-1ELISA Kit

The Sirtuin 1 (Sirt-1) protein was quantified with the Human SIRT1 ELISA kit (Thermo Scientific^TM^, Waltham, MA, USA) on the SH-SY5Y lysate. Briefly, 100 μL of standard or sample was added to each well, and the plate was incubated at room temperature for 2.5 h. After incubation, the wells were rinsed with 1× wash buffer, and 100 µL of biotin-conjugate was added. The mixture was then incubated for 1 h at room temperature. Each well was rewashed with 1× wash buffer, and 100 μL of Streptavidin-HRP was added. The plate was incubated at room temperature for 45 min before adding 100 μL of TMB Substrate to each well and incubating for 30 min in the dark. After adding 50 μL of stop solution to each well, the absorbance was measured at 450 nm using a spectrometer (Infinite 200 Pro-MPlex, Tecan, Männedorf, Switzerland). The results are presented as a percentage (%) of the control (line 0, untreated cells) from five separate experiments, each carried out in triplicate. The concentration is reported as ng/mL on a standard curve (range: 1.23 to 300 ng/mL).

### 2.25. pTAU ELISA Kit

Quantification of TAU protein was measured with the Human Tau ELISA kit [pS199] (Thermo Scientific^TM^, Waltham, MA, USA) on the SH-SY5Y lysate. Briefly, 100 μL of standard or sample was added to each well, and the plate was incubated at room temperature for 2 h. After incubation, the wells were rinsed with 1× wash buffer, and 100 μL of anti-Rabbit IgG HRP solution was added. The mixture was then incubated at room temperature for 30 min. Each well received 100 μL of stabilised chromogen after being rewashed with 1× wash buffer. After 30 min of incubation at room temperature, 100 μL of stop solution was added to each well of the plate. A spectrometer (Infinite 200 Pro-MPlex, Tecan, Männedorf, Switzerland) detected the absorbance at 450 nm. The concentration was then quantified in pg/mL compared to a standard curve ranging from 15.6 to 1000 pg/mL. The findings are presented as a percentage (%) to the control (line 0, untreated cells) of five separate, triplicate tests.

### 2.26. NRF2 ELISA Kit

The NRF2 Quantification ELISA kit (MyBiosource, San Diego, CA, USA) was used to measure the presence of Nrf2 in SH-SY5Y lysates, following the manufacturer’s instructions. Briefly, A 96-well plate was filled with 100 μL of each sample, then incubated for 90 min at 37 °C. The material was then removed, and each well was cleaned three times. We added 100 μL of detection solution A and incubated for 45 min at 37 °C before cleaning the wells and adding 100 μL of detection solution B. The plate was incubated at 37 °C for 45 min. After adding 90 μL of substrate solution to each well, the plate was incubated in the dark at 37 °C for 20 min. After stopping the reaction with 50 μL of stop solution, the absorbance was measured at 450 nm using a spectrometer (Infinite 200 Pro-MPlex, Tecan, Männedorf, Switzerland). By comparing the findings with the standard curve (0–25 ng/mL) of five separate triplicate experiments, the concentration is expressed in ng/mL.

### 2.27. Statistical Analysis

The statistical software GraphPad 9.4.1 (GraphPad Software, La Jolla, CA, USA) was used to analyse the data, which came from at least five separate experiments carried out in triplicate for each experimental protocol. The results are presented as mean ± SD (standard deviation) for statistical analysis using a one-way ANOVA and the Bonferroni post hoc test. Statistical significance was defined as a *p*-value of less than 0.05.

## 3. Results

### 3.1. The Dose–Response and Time-Course Effects of the Substances Selected at the Intestinal Level

**Figure 2:**
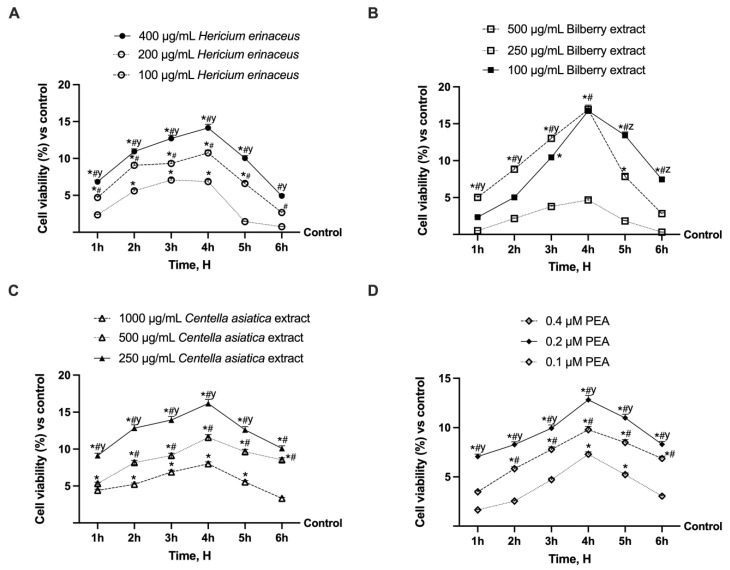
Time-course and dose–response analysis of Caco-2 cell viability. In (**A**) *Hericium erinaceus*, * *p* < 0.05 vs. control; # *p* < 0.05 vs. 200 µg/mL *Hericium erinaceus*; y *p* < 0.05 vs. 100 µg/mL *Hericium erinaceus*. In (**B**), Bilberry extract, * *p* < 0.05 vs. control; # *p* < 0.05 vs. 250 µg/mL Bilberry extract; y *p* < 0.05 vs. 100 µg/mL Bilberry extract; z *p* < 0.05 vs. 500 µg/mL Bilberry extract. In (**C**), *Centella asiatica* extract, * *p* < 0.05 vs. control; # *p* < 0.05 vs. 1000 µg/mL *Centella asiatica* extract; y *p* < 0.05 vs. 500 µg/mL *Centella asiatica* extract. In (**D**), PEA, * *p* < 0.05 vs. control; # *p* < 0.05 vs. 0.1 µM PEA; y *p* < 0.05 vs. 0.4 µM PEA. Compared to the control values (0% line), the data are presented as the mean (%) ± SD of five experiments conducted in triplicate.

To assess the optimal concentration of each selected substance and prevent any cytotoxic effects, the cell viability of Caco-2 cells was analysed from 1 h to 6 h using MTT tests. As shown in Figure 2, the concentrations of all substances tested showed increased cell viability compared to the control from 3 h of treatment (*p* < 0.05). The 400 µg/mL concentration of *Hericium erinaceus* exerted a significant effect compared to the other two concentrations tested for all treatment times (Figure 2A). The maximum effect was observed at 4 h of treatment, with increases of approximately 51% and 24% compared to the 200 µg/mL and 100 µg/mL concentrations, respectively (*p* < 0.05). In the case of Bilberry extract (Figure 2B), the highest and lowest concentrations selected proved to be the best (*p* < 0.05) with an almost identical effect at 4 h of treatment with an increase over the 250 µg/mL concentration of around 72% (*p* < 0.05); however, the lower one (100 µg/mL) was selected following the pharmacodynamic principle that the best choice is the lowest dose with the most significant effect. In the case of *Centella asiatica* extract, the concentration with the best effect across all treatment times (*p* < 0.05) was the lowest selected, 250 µg/mL. The 250 µg/mL *Centella asiatica* extract showed a peak at 4 h of treatment, associated with the greatest effect compared with the other two concentrations of 1000 µg/mL and 500 µg/mL, by approximately 50.5% and 28.4%, respectively (Figure 2C, *p* < 0.05). As shown in Figure 2D, as a result of dose–response screening also for PEA, one concentration with the best effect, which is the 0.2 µM, has selected, with a significant effect on the other two chosen for all times examined (*p* < 0.05); again, the peak effect was observed at 4h of treatment with a more significant effect of around 24% and 43% compared to the 0.4 µM and 0.1 µM concentrations (Figure 2D, *p* < 0.05). To summarise, the chosen concentrations include 400 μg/mL *Hericium erinaceus*, 100 µg/mL Bilberry extract, 250 μg/mL *Centella asiatica*, and 0.2 μM of PEA.

### 3.2. Analysis of the Effects at the Intestinal Level to Explore Integrity and Absorption Through an Intestinal Barrier Model

Before examining the impact of the substances, individually and in combination, on neural tissue, experimental procedures were conducted to evaluate the influence of these substances on a rigorously validated in vitro 3D intestinal barrier model [61,62,82]. The experiments on this model enabled the evaluation of cell viability and the integrity of the intestinal barrier by recording TEER values throughout a treatment duration ranging from 1 h to 6 h and by analysing the concentrations of TJ proteins following the conclusion of the 6 h treatment. TJs, such as claudin-1, occludin, and ZO-1, represent the multiprotein junctional complex that has a regulatory function in the passage of ions, water and solutes through the paracellular pathway, thus also regulating the function of the intestinal barrier [83].

**Figure 3:**
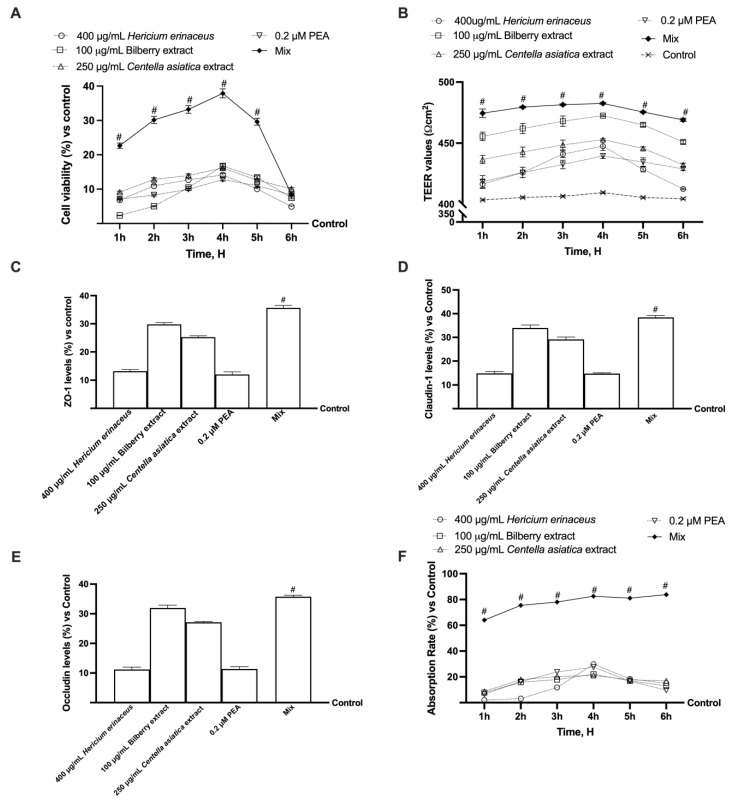
A model of the intestinal barrier in vitro. Cell viability analysis of the combination, all the agents *p* < 0.05 vs. control except 400 μg/mL *Hericium erinaceus* at 6 h and 100 µg/mL Bilberry extract a 1h, # *p* < 0.05 vs. single agents (**A**); the TEER value is assessed using EVOM3, all the agents *p* < 0.05 vs. control except 400 μg/mL *Hericium erinaceus* at 6 h, # *p* < 0.05 vs. single agents (**B**); the study of tight junctions is conducted via ELISA tests for claudin-1, occludin, and ZO-1, all the agents *p* < 0.05 vs. control, # *p* < 0.05 vs. single agents (**C**–**E**); the evaluation of passage through the intestinal barrier is performed using a fluorescent trace, all the agents *p* < 0.05 vs. control except 400 μg/mL *Hericium erinaceus* at 1 h and 2 h, # *p* < 0.05 vs. single agents (**F**). Five separate tests were conducted in triplicate, and the results are presented as means ± SD (%), compared to the control values (the 0% line). Mix = 400 μg/mL *Hericium erinaceus* + 100 µg/mL Bilberry extract +250 μg/mL *Centella asiatica* + 0.2 μM of PEA.

After selecting the concentrations, their combination was evaluated. As illustrated in Figure 3A, the concentrations demonstrate a positive influence on cell viability compared to the control throughout the treatment duration, and this effect is further enhanced when combined (*p* < 0.05). The results obtained demonstrated that the combination (referred to as Mix) boosts cell viability by approximately 63% (vs. 400 µg/mL *Hericium erinaceus*, *p* < 0.05), 56% (vs. 100 µg/mL Bilberry extract, *p* < 0.05), 57% (vs. 250 µg/mL *Centella asiatica*, *p* < 0.05), and 66% (vs. to 0.4 µM PEA). As illustrated in Figure 3B, the intestinal epithelium analysis also demonstrated that all tested substances could preserve epithelial integrity compared to the control (*p* < 0.05) by increasing the ion flux of paracellular exchanges across the intestinal barrier. The best effect was obtained following stimulation with the combination during all stimulation times compared to the individual agents tested (*p* < 0.05). Mix exhibited its highest effect at 4 h, allowing a percentage increase of about 5%, 9%, 10%, and 12% compared to 100 µg/mL Bilberry extract, 250 µg/mL *Centella asiatica*, 400 µg/mL *Hericium erinaceus*, and 0.2 µM PEA, respectively (*p* < 0.05). The analysis of TJs also confirmed the results obtained from the TEER analysis. In particular, occludin, which contributes to the stabilisation and optimal function of the barrier, claudin-1, the main barrier-forming protein, and ZO-1, which maintains and modulates barrier integrity simultaneously, were analysed. The results shown in Figure 3C–E demonstrated that the individual agents were able to increase the levels of claudin-1, occludin, and ZO-1 compared to the control (400 µg/mL *Hericium erinaceus* about 14.8%, 11.2%, and 13.1%, *p* < 0.05; 100 µg/mL Bilberry extract about 34%, 32%, and 29.7%, *p* < 0.05; 250 µg/mL *Centella asiatica* about 29.1%, 27.1%, and 23.3%, *p* < 0.05; 0.2 µM PEA 14.7%, 11.3%, and 12%, *p* < 0.05). The effect was amplified following treatment with Mix, which was able to increase the levels of claudin-1 (about 61% vs. 400 µg/mL *Hericium erinaceus*, about 11.5% vs. 100 µg/mL Bilberry extract, about 24.2% vs. 250 µg/mL *Centella asiatica*, and about 62% vs. 0.2 µM PEA, *p* < 0.05), occludin (about 69% vs. 400 µg/mL *Hericium erinaceus*, about 10.6% vs. 100 µg/mL Bilberry extract, about 24% vs. 250 µg/mL *Centella asiatica*, and about 68% vs. 0.2 µM PEA, *p* < 0.05), and ZO-1 (about 63% vs. 400 µg/mL *Hericium erinaceus*, about 16.4% vs. 100 µg/mL Bilberry extract, about 29.2% vs. 250 µg/mL *Centella asiatica*, and about 66.4% vs. 0.2 µM PEA, *p* < 0.05). The absorption rate was also analysed using a fluorescent probe to complete the picture of the intestinal analysis. As shown in Figure 3F, Mix consistently demonstrated a significantly higher absorption level compared to the individual agents (*p* < 0.05).

### 3.3. The Effect of the Substances on the Gut–Brain Axis Model

**Figure 4:**
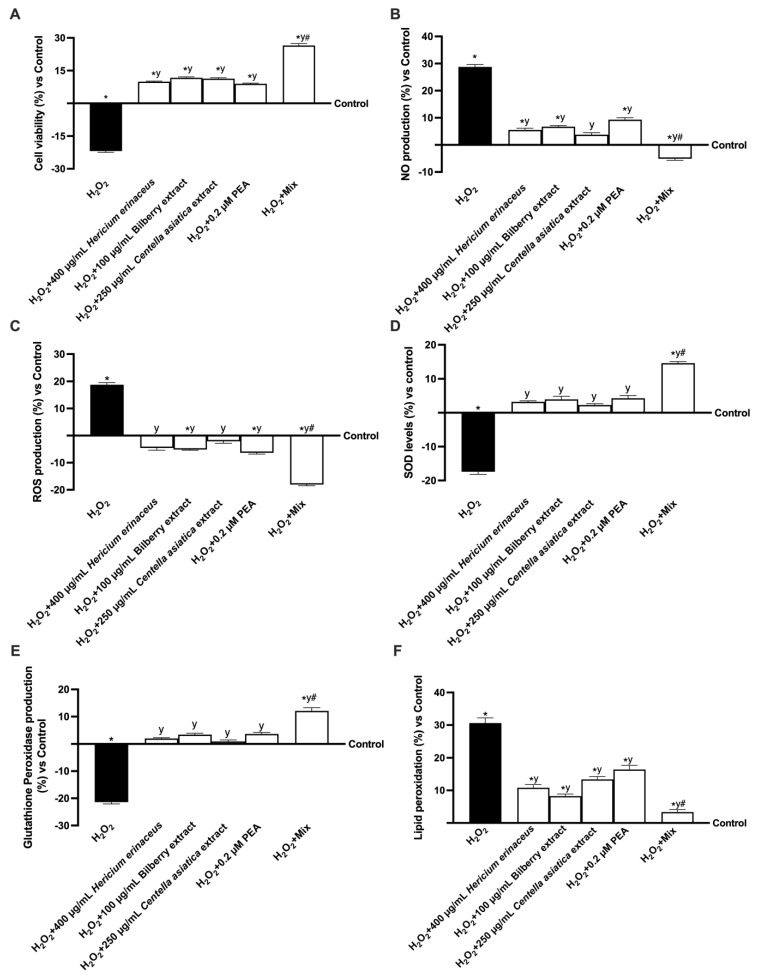
Evaluation of the effects on the gut–brain axis under oxidative stress. In (**A**) cell viability analysis by MTT test; in (**B**) NO production analysis; in (**C**) ROS production analysis measured through cytochrome C reduction; in (**D**) SOD level analysis measured through ELISA kit; in (**E**) GPx production analysis; in (**F**) lipid peroxidation analysis. Data from five experiments, performed in triplicate, are presented as averages ± SD (%) and compared to the control values (0% line). The abbreviations are the same as Figure 3. * *p* < 0.05 vs. control; y *p* < 0.05 vs. 200 µM H_2_O_2_; # *p* < 0.05 vs. single agents.

To replicate cognitive decline in vitro, a gut–brain axis model was pre-exposed to 200 µM H_2_O_2_ to induce oxidative stress, followed by treatment with individual compounds and their combination; subsequent analyses assessed cell viability, reactive oxygen species generation, nitric oxide production, and lipid peroxidation rate (Figure 4). As shown in Figure 4A, treatment with 200 µM H_2_O_2_ alone significantly reduced cell viability (about 21.9% vs. control, *p* < 0.05), SOD levels (about −17% vs. control, *p* < 0.05), inducing a simultaneous GPx production (about −21% vs. control, *p* < 0.05), ROS and NO productions (about 18.75% and 28.78%, respectively, *p* < 0.05) and lipid peroxidation increased (about 30.6% compared to control, *p* < 0.05). In contrast, the adverse conditions were significantly counteracted by the presence of all substances tested individually and in combination (*p* < 0.05). The combination tested improved cell viability, SOD levels, and GPx production, while simultaneously reducing ROS and NO production and lipid peroxidation compared to the control (*p* < 0.05) and compared to H_2_O_2_-induced damage (*p* < 0.05). The combination had the most significant effect on individual substances (*p* < 0.05), supporting the hypothesis of the efficacy of this combination. Specifically, Mix increased cell viability after damage by approximately 62%, 56%, 57%, and 66% compared to 400 µg/mL *Hericium erinaceus*, 100 µg/mL Bilberry extract, 250 µg/mL *Centella asiatica*, and 0.2 µM PEA. Further Mix induced an increase in SOD levels and GPx production of approximately 3.5-fold and 4.9-fold, 2.7-fold and 2.5-fold, 4.4-fold and 12-fold, and 2.4-fold and 2.3-fold compared to 400 µg/mL *Hericium erinaceus*, 100 µg/mL Bilberry extract, 250 µg/mL *Centella asiatica*, and 0.2 µM PEA. In comparison, it reduced ROS and NO production by approximately 74% and 2-fold, 71% and 2.3-fold, 88% and 1.7-fold, and 65% and 2.8-fold compared to 400 µg/mL *Hericium erinaceus*, 100 µg/mL Bilberry extract, 250 µg/mL *Centella asiatica*, and 0.2 µM PEA, respectively. Finally, Mix also reduced lipid peroxidation compared to the individual agents by approximately 2.2-fold vs. 400 µg/mL *Hericium erinaceus* (*p* < 0.05), 1.47-fold vs. 100 µg/mL Bilberry extract (*p* < 0.05), 3-fold vs. 250 µg/mL *Centella asiatica* (*p* < 0.05), and 3.9-fold vs. 0.2 µM PEA (*p* < 0.05).

**Figure 5:**
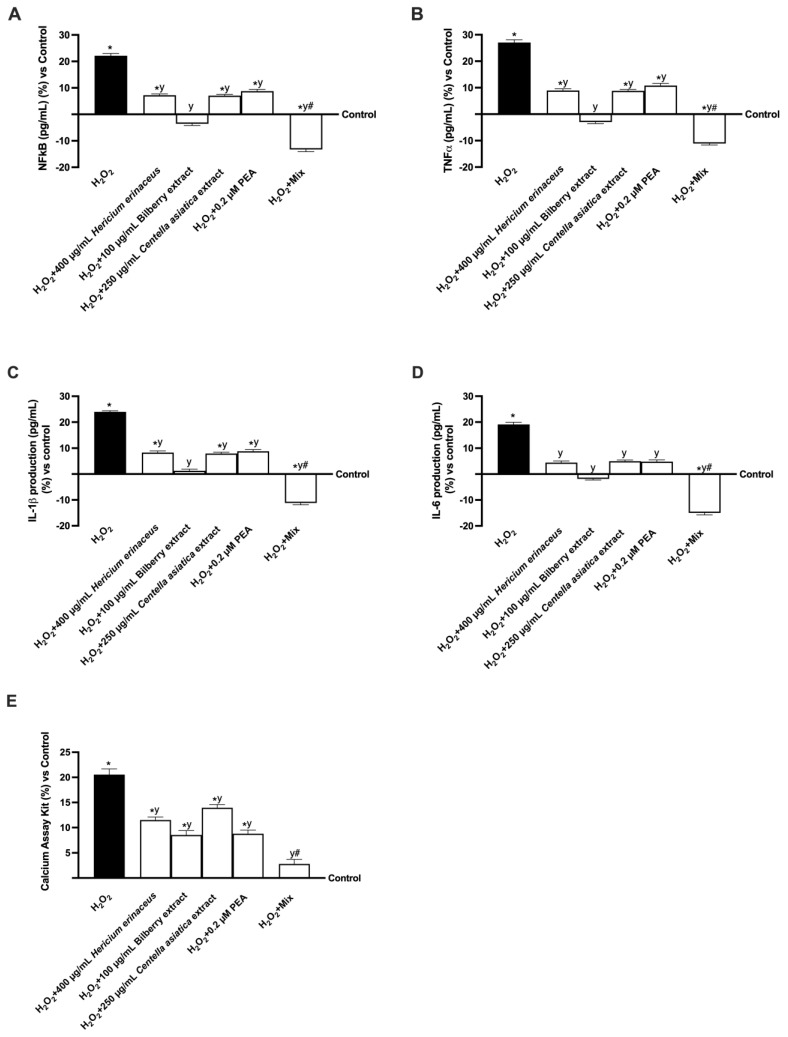
Analysis of inflammatory processes and calcium levels. In (**A**) TNFα quantification analysis, (**B**) NFkB quantification analysis, (**C**) IL-1β quantification analysis, and (**D**) IL-6 quantification analysis by ELISA Kit; in (**E**) calcium quantification analysis. All the analyses were performed using a specific kit. Data from five experiments in triplicate are presented as averages ± SD (%) and compared to the control values (0% line). The abbreviations are the same as Figure 3. * *p* < 0.05 vs. control; y *p* < 0.05 vs. 200 µM H_2_O_2_; # *p* < 0.05 vs. single agents.

Given the link between oxidative stress, inflammation, and cognitive decline, additional analyses were conducted to assess key inflammatory markers involved in this process. Therefore, TNFα and NF-kB were selected as markers of inflammation and investigated by mimicking the condition of cognitive decline in vitro by treatment with 200 μM H_2_O_2_ (Figure 5). Specifically, as shown in Figure 4B and Figure 5A, the harmful activity induced by 200 μM H_2_O_2_ resulted in approximately 27% and 22% increases in TNFα and NF-κB levels, respectively, compared to the control (*p* < 0.05). In contrast, treatment with the individual agents and their combination reduced this condition (*p* < 0.05). More precisely, all single extracts were able to reduce the levels of inflammatory cytokines TNFα and NF-kB by about 67% and 67.3% (400 µg/mL *Hericium erinaceus* vs. 200 µM H_2_O_2_, *p* < 0.05), 1.1-fold and 1.2-fold (100 µg/mL Bilberry extract vs. 200 µM H_2_O_2_, *p* < 0.05), 67.5% and 68% (250 µg/mL *Centella asiatica* vs. 200 µM H_2_O_2_, *p* < 0.05), and 60% and 60.3% (0.2 µM PEA vs. 200 µM H_2_O_2_, *p* < 0.05). In addition, the effects were more significant once the individual extracts were combined: Mix exerted more significant beneficial effects in terms of reduction both to H_2_O_2_-induced damage (1.4-fold and 1.6-fold, TNFα and NF-kB, respectively, *p* < 0.05) and compared to single agents (1.9-fold and 1.6-fold vs. 400 µg/mL *Hericium erinaceus*; 73% and 73.1% vs. 100 µg/mL Bilberry extract; 1.8-fold and 1.5-fold vs. 250 µg/mL *Centella asiatica*; and 2-fold and 1.7-fold vs. 0.2 µM PEA, TNFα and NF-kB, respectively, *p* < 0.05). Two interleukins were also evaluated to provide a more comprehensive profile of the inflammatory response. As expected, 200 μM H_2_O_2_ caused an increase in levels of IL-1β and IL-6 of approximately 24% and about 19%, respectively, compared to the control (*p* < 0.05). Even in this case, the negative condition was reduced by the individual agents and their combination (*p* < 0.05). All single extracts were able to reduce the levels of IL-1β and IL-6 compared to 200μM H_2_O_2_ (*p* < 0.05). Again, the effects were more significant with the combination: Mix induced a decrease in IL-1β and IL-6 compared to H_2_O_2_-induced damage (1.5-fold and 1.8-fold, respectively, *p* < 0.05) and compared the single agents by about 2.4-fold and 4.4-fold (400 µg/mL *Hericium erinaceus*, *p* < 0.05), 9.5-fold 1 and 7.3-fold (100 µg/mL Bilberry extract, *p* < 0.05), 2.4-fold and 4-fold (250 µg/mL *Centella asiatica*, *p* < 0.05), and 2.3-fold and 4.1-fold (0.2 µM PEA, *p* < 0.05). Inflammation in the brain, which can lead to cognitive loss and the advancement of neurodegenerative illnesses, can be impacted by calcium level dysregulation. For this reason, the final analysis performed on the in vitro gut–brain axis model focused on measuring calcium levels. As shown in Figure 5E, pretreatment with 200 μM H_2_O_2_ resulted in approximately a 21% increase in calcium levels compared to the control (*p* < 0.05), indicating that in the damaged condition, calcium is accumulated within the cells. On the other hand, treatment with the single agents reduced this condition (*p* < 0.05). All the extracts were able to reduce the levels of calcium compared to H_2_O_2_ by about 78% (400 µg/mL *Hericium erinaceus*, *p* < 0.05), 1.4-fold (100 µg/mL Bilberry extract, *p* < 0.05), 47% (250 µg/mL *Centella asiatica*, *p* < 0.05), and 1.3-fold (0.2 µM PEA, *p* < 0.05). Also in this case, the effects were more significant with the combination: Mix exerted a strong, significant reduction of calcium levels both compared to 200 µM H_2_O_2_ (6.4-fold, *p* < 0.05) and compared to single agents (75% vs. 400 µg/mL *Hericium erinaceus*; 67% vs. 100 µg/mL Bilberry extract; 80% vs. 250 µg/mL *Centella asiatica*; and 68% vs. 0.2 µM PEA, *p* < 0.05).

### 3.4. The Effect of the Substances on a Co-Culture of Sh-Sy5y and Ccf-Sttg1 Cells

**Figure 6:**
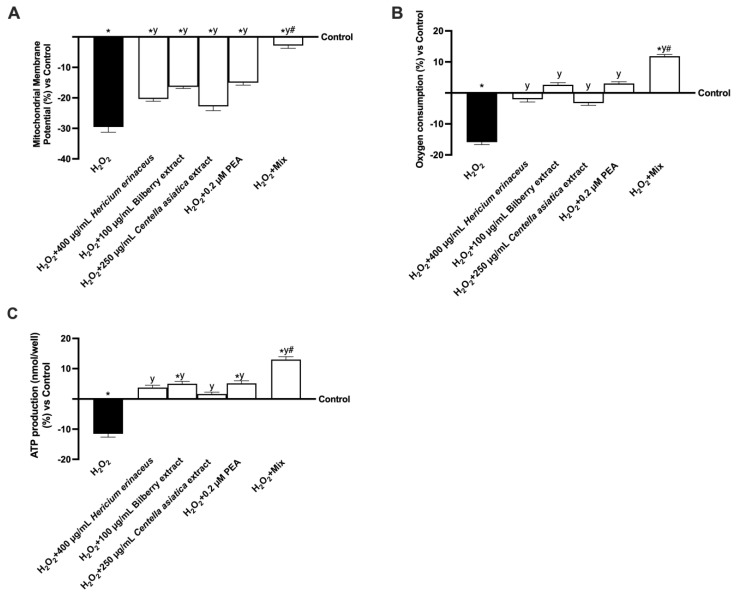
Analysis of cellular energy. In (**A**), mitochondrial potential membrane analysis; in (**B**), oxygen consumption analysis; in (**C**) ATP level analysis. All the analyses were performed using the ELISA test. The data are reported as means ± SD (%) of five independent experiments performed in triplicate and compared to the control values (the 0% line). The abbreviations are the same as Figure 3. * *p* < 0.05 vs. control; y *p* < 0.05 vs. 200 µM H_2_O_2_; # *p* < 0.05 vs. single agents.

In addition to oxidative stress and inflammation, cellular energy metabolism plays a crucial role in maintaining cognitive function. To explore this aspect in the context of cognitive decline, further analyses were conducted on a co-culture of SH-SY5Y and CCF-STTG1 cells. Firstly, JC-1 staining revealed that all individual compounds significantly influenced mitochondrial activity, particularly by enhancing mitochondrial membrane potential. As shown in Figure 6A, all agents were able to produce an increase in potential compared to 200 μM H_2_O_2_ (*p* < 0.05), indicating a physiological rise in the cells’ chemical energy. Once more, Mix induced the most significant effects: Mix induced an increase compared to H_2_O_2_-induced damage (about 90%, *p* < 0.05) and about 86% compared to 400 µg/mL *Hericium erinaceus*, 82% compared to 100 µg/mL Bilberry extract, 87% compared to 250 µg/mL *Centella asiatica*, and 81% compared to 0.2 µM PEA *(p* < 0.05). Oxygen consumption is closely related to membrane potential, particularly in the context of mitochondrial function. As shown in Figure 6B, 200 μM H_2_O_2_ induced a significant decrease in oxygen consumption compared to the control (*p* < 0.05). All single agents were able to restore this condition compared to 200 μM H_2_O_2_ (*p* < 0.05), bringing the levels almost back to the control value. Again, Mix induced an increase compared to H_2_O_2_-induced damage (about 1.8-fold, *p* < 0.05) and about 6.9-fold compared to 400 µg/mL *Hericium erinaceus*, 3.6-fold compared to 100 µg/mL Bilberry extract, 4.6-fold compared to 250 µg/mL *Centella asiatica*, and 2.9-fold compared to 0.2 µM PEA *(p* < 0.05). In turn, oxygen consumption is closely related to the production of ATP. ATP production analyses (Figure 6C) are consistent with those of the first two analyses. 200 μM H_2_O_2_ induced a significant decrease in ATP production compared to the control (*p* < 0.05). At the same time, production was increased after treatment with all single agents, compared not only to 200 μM H_2_O_2_ (*p* < 0.05) but also to the control. However, only 100 µg/mL Bilberry extract induced a significant increase in ATP production versus the control (*p* < 0.05). Once more, their combined use significantly outperformed the impact of individual agents. Indeed, Mix induced an increase in ATP production of about 2.4-fold compared to 400 µg/mL *Hericium erinaceus*, 6.6-fold compared to 100 µg/mL Bilberry extract, 4.6-fold compared to 250 µg/mL *Centella asiatica*, and 1.5-fold compared to 0.2 µM PEA *(p* < 0.05).

**Figure 7:**
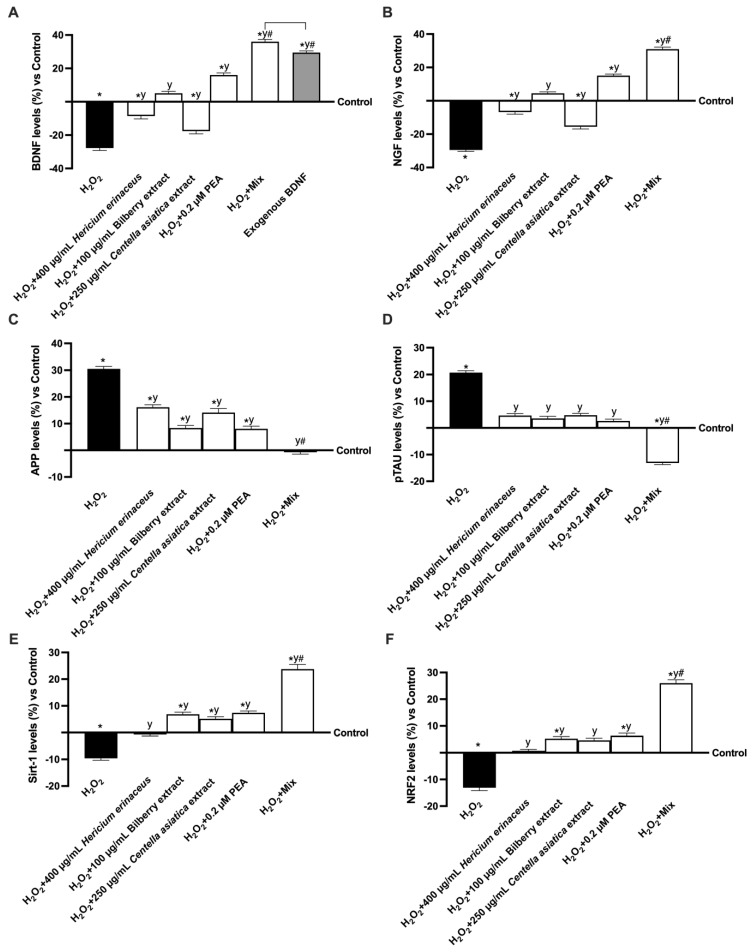
Evaluation of neurotrophins involved in cognitive decline in vitro. In (**A**), BDNF quantification, α *p* < 0.05 vs. control; β *p* < 0.05 vs. 200 µM H_2_O_2_; γ *p* < 0.05 vs. single agents; δ *p* < 0.05 vs. exogenous BDNF (use concentration 10 ng/mL); in (**B**) NGF quantification; in (**C**) APP quantification; in (**D**) pTAU quantification; in (**E**) Sirt-1 quantification; in (**F**) NRF2 quantification. All the analyses were performed using the ELISA test. The data are reported as means ± SD (%) of five independent experiments performed in triplicate and compared to the control values (represented by the 0% line). The abbreviations are the same as Figure 3. * *p* < 0.05 vs. control; y *p* < 0.05 vs. 200 µM H_2_O_2_; # *p* < 0.05 vs. single agents; the bar *p* < 0.05 vs. exogenous BDNF.

Considering the range of effects that neurotrophins such as BDNF and NGF have on neuronal function, their activity in the context of cognitive decline was analysed in vitro (Figure 7A,B). As expected, treatment with only 200 µM H_2_O_2_ resulted in a decrease in the production of these two neurotrophins (approximately 25.1% and 27.9%, respectively, *p* < 0.05), consistent with the effects observed in the presence of cognitive decline. Regarding the substances examined, all of them were able to increase the levels of BDNF and NGF compared to 200 µM H_2_O_2_ damage; especially, the levels of BDNF and NGF were increased by about 64% and 70% (400 µg/mL *Hericium erinaceus* vs. 200 µM H_2_O_2_, *p* < 0.05), 1.15-fold and 1.1-fold (100 µg/mL Bilberry extract vs. 200 µM H_2_O_2_, *p* < 0.05), 21% and 39% (250 µg/mL *Centella asiatica* vs. 200 µM H_2_O_2_, *p* < 0.05), and 1.6-fold and 1.5-fold (0.2 µM PEA vs. 200 µM H_2_O_2_, *p* < 0.05). In conclusion, Mix was able to exert a beneficial effect on BDNF production compared to the endogenous BDNF sample, increasing this production by approximately 19% (*p* < 0.05). More in detail, Mix increases BDNF and NGF levels more effectively than damage and single agents, confirming the beneficial effects of this combination (about 2.3-fold and 2-fold vs. 200 µM H_2_O_2_; about 5.2-fold and 5.4-fold vs. 400 µg/mL *Hericium erinaceus*; about 6.2-fold and 5.9-fold vs. 100 µg/mL Bilberry extract; about 3.1-fold and 3-fold vs. 250 µg/mL *Centella asiatica*; and about 1.25-fold and 1.1-fold vs. 0.2 µM PEA, *p* < 0.05).

Finally, the principal intracellular pathways involved in cognitive decline were analysed by mimicking the damage condition with 200 µM H_2_O_2_. In detail, to assess the effect of the test substances on brain trophism, the levels of APP, a beta-amyloid precursor, and pTAU, a protein involved in cognitive deterioration, were analysed (Figure 7C,D). The levels of both proteins increased following treatment with 200µM H_2_O_2_, thus demonstrating their involvement in the context of cognitive decline, with an increase in APP and pTAU levels of approximately 28% and 19.1%, respectively, compared to control (*p* < 0.05). In contrast, treatment with the single agents reduced APP and pTAU levels, thereby demonstrating an improvement over damage-induced levels (*p* < 0.05). Specifically, Mix reduced APP and pTAU levels more effectively than damage and single agents, confirming the cooperative ability of the single agents when combined (about 97% and 1.63-fold vs. 200 µM H_2_O_2_; about 96% and 1.5-fold vs. 400 µg/mL *Hericium erinaceus*; about 94% and 1.4-fold vs. 100 µg/mL Bilberry extract; about 96% and 1.52-fold vs. 250 µg/mL *Centella asiatica*; and about 93% and 1.28-fold vs. 0.2 µM PEA, *p* < 0.05). To further confirm the data obtained, the levels of Sirt-1, a protein responsible for neuronal energy metabolism, and the levels of NRF2, a molecule involved in protecting cognitive function, were analysed (Figure 7E,F). All the substances increased the levels of both proteins, restoring physiological levels or even more significantly (*p* < 0.05). Specifically, Mix increased Sirt-1 and NRF2 levels more effectively than damage and single agents by about 1.4-fold and 1.5-fold vs. 200 µM H_2_O_2_ (*p* < 0.05), by about 1.1-fold and 1.05% vs. 400 µg/mL *Hericium erinaceus* (*p* < 0.05), by about 73% and 82% vs. 100 µg/mL Bilberry extract (*p* < 0.05), by about 82% and 86% vs. 250 µg/mL *Centella asiatica* (*p* < 0.05), and by about 74% and 79% vs. 0.2 µM PEA (*p* < 0.05).

## 4. Discussion

Bioactive substances have been studied for their potential to prevent cognitive decline and improve cognitive abilities [11]. Polyphenols can modify brain function at three levels: externally to the CNS, within the CNS, and at the blood–brain barrier (BBB) level. Neurotrophin reduction, including BDNF, is involved in developing various CNS diseases, including neurodegenerative and psychiatric disorders [16].

The study, therefore, aimed to analyse the modulation of two main neurotrophins and key markers associated with cognitive decline conditions. The first step was to find the ideal concentration of each component to preserve its properties. Focusing on intestinal activity, during the second phase of analysis, the absence of side effects related to oral consumption of the nutraceutical component under consideration was confirmed. The elevated Intestinal TJ levels, “essential metrics” for assessing an epithelial monolayer setup in vitro, further supported this. Studies of claudin-1, occludin, and ZO-1 validate appropriate gut function. Furthermore, TEER research supports the active involvement of all the substances. This information was validated by a high absorption rate and analysed through a fluorescent probe, indicating the possibility of a synergistic effect between the individual components.

In the third part of the study, we evaluated several nutraceuticals using an in vitro model of cognitive decline, which reproduces the gut–brain axis through Transwell absorption along the intestinal barrier and subsequent passage to the nerve target. The research focused on ROS and NO production to understand their ability to attenuate oxidative stress. Ageing is associated with oxidative stress, characterised by increased levels of ROS and malondialdehyde (MDA), a marker of lipid peroxidation, which impairs cellular function [84,85]. The study found that when combined, *Hericium erinaceus*, PEA, Bilberry extract, and *Centella asiatica* restrained and reduced MDA production, thereby decreasing lipid peroxidation levels.

The data obtained can be linked to the presence of polyphenols in *Hericium erinaceus*, Bilberry extract, and *Centella asiatica*, as their antioxidant activity allows them to act as reducing agents, hydrogen donors, singlet oxygen quenchers and metal chelators [86]. Indeed, the composition of these extracts (Table A1), including β-glucans on *Hericium erinaceus* and anthocyanins on Bilberry extract, has been crucial in determining their antioxidant capabilities. The polysaccharide content (14% of β-1,3-glucans) has lowered ROS levels and enhanced the overall oxidative environment, promoting cell survival in stressed nerve cells. The ability of polysaccharides to alter cellular signals linked to inflammation and apoptosis is also considered, in part, due to their antioxidant capabilities [87,88]. Bilberry extract’s bioactive components, including delphinidin, cyanidin, peonidin, and petunidin, have been found to have a relationship with neuronal effects. Anthocyanins, which contain hydroxyl groups linked to aromatic compounds, neutralise ROS and reduce oxidative damage in vitro [70,89]. On the other hand, PEA does not contain polyphenols, but its anti-inflammatory action, combined with antioxidants, can potentiate its pharmacological effects. Indeed, the combination strongly decreased oxidative stress, demonstrating the synergistic activity of the substances.

The scientific literature has also demonstrated a critical connection between systemic inflammation and cognitive disorders, as well as a strong association between oxidative stress and neurodegeneration [90,91]. As we age, inflammation levels increase, leading to the production of pro-inflammatory cytokines, such as TNF-α [92]. Neuroinflammation impairs the structure and function of the brain. Nutraceutical tests have shown a decrease in inflammatory markers in a condition that mimics cognitive impairment. The mixed treatment has been found to significantly reduce TNFα and NF-κB levels. The major activity of the Mix can be attributed to the composition of the single substances chosen. Indeed, the delphinidins and other anthocyanins (cyanidin, petunidin and peonidin) in Bilberry extract can modulate neuroinflammation-related signalling pathways, promoting regeneration of synaptic connections [70]; while the antioxidant properties of *Hericium erinaceus* extract with the presence of β-1,3-glucans have modulated cellular signals related to inflammation and apoptosis, as reported in the literature [89]. In this regard, it is important to reiterate the choice of *Centella asiatica* because its essential constituents, including madecassic acid, aspartic acid, and asiaticoside, have a variety of anti-inflammatory and, consequently, neuroprotective properties [93]. The selected extract of *Centella asiatica* used in the study has a good concentration of asiaticosides and the presence of madecassoside, free flavonoids and dicaffeoylquinic acid. These components, both alone and in combination, have been extensively researched for their neuroprotective and antioxidant properties, particularly in models of neurodegeneration and cognitive decline [94]. The extract was tested in the literature on cellular models such as SH-SY5Y cells, which mimic oxidative stress and neural inflammation [94]. Asiaticoside was found to increase synaptic plasticity and reduce inflammation in neurodegenerative disorders. Bilberry also reduces inflammation by modifying NFkB signalling pathways and lowering levels of inflammatory cytokines such as TNF-α, IL-1β and IL-6 [95,96]. Known for its anti-inflammatory properties, PEA was also used to explain the selection of combinations. The results are predictable and provide insight into the potential benefits of PEA in terms of neuroprotective and antioxidant properties [97]. Thus, every substance is characterised by a slightly different activity on the pathways regarding inflammation, and this is the reason for the more positive results obtained from the combination.

It is widely recognised that calcium overload in neuronal cells contributes significantly to the progression of neuronal pathologies such as Alzheimer’s disease. Indeed, calcium excess disrupts brain network functions, contributing to synaptic and cognitive deficiencies [98]. Thus, the ability of the formulation under examination to lower calcium levels in neuronal cells to values comparable to those of the control assumes great importance. From this data, it is possible to assume that the combination of *Hericium erinaceus*, PEA, Bilberry extract, and *Centella asiatica* could effectively deplete calcium accumulation in neuronal cells.

Complications with energy metabolism can also influence the complex process of brain ageing. Indeed, the areas most susceptible to energy deficits, such as the cerebral cortex, the hippocampus, and the basal ganglia, as well as certain functions like memory, cognition, and motor control, are where the impacts of ageing are most noticeable [99]. This study demonstrated that treatment with a combination of *Hericium erinaceus*, PEA, Bilberry extract, and *Centella asiatica* completely restored the levels of membrane potential, oxygen consumption, and ATP production, all of which are biological markers involved in energetic metabolism in a co-culture of SH-SY5Y and CCF-STTG1 cells.

The study also found that in the co-culture, BDNF and NGF levels increased after treatment with the test substances, suggesting the potential of the formulation in promoting neurogenesis and its utility in cognitive decline. Neurotrophins play a crucial role in the proliferation, differentiation and durability of neurons in the PNS and CNS [100]. The results indicate that the combination of *Hericium erinaceus*, PEA, Bilberry extract, and *Centella asiatica* promotes BDNF-triggered and regulated neurogenesis. It is already known that through the action of hericenones (C, D, E, H) and erinacines (A-F, H), *Hericium erinaceus* can stimulate the production of NGF and that erinacine C also increases the expression of BDNF [26]. Furthermore, erinacine A acts at the neurotransmitter-neurotrophin level, inducing neurotrophin synthesis by regulating noradrenaline [101]. On the other hand, Bilberry is known to induce an increase in BDNF through the regulation of protein translation [102]. Further, glutamate, an amino acid found in *Centella asiatica*, is a precursor to gamma-aminobutyric acid (GABA), which is thought to function as an excitatory neurotransmitter that can raise BDNF expression via the mitogen-activated protein kinase (MAPK)-acyl response element-binding protein (CREB) pathway [103]. Finally, PEA is also known to regulate neurotrophin production through CREB regulation [104]. Thus, in this case, the major effects of the combination on neurotrophin production could be due to the similar action of multiple extracts.

The accumulation of β-amyloid and pTAU correlated with mild cognitive impairment [105] was found to be significantly reduced after treatment. Indeed, a cognitive decline condition in vitro increased APP and pTAU levels. In contrast, this condition was strongly decreased following treatment, confirming the beneficial effect of the formulation under investigation. Finally, Sirt-1 and NRF2 were analysed to complete the neuronal framework analysis. Upstream of Sirt-1, NRF2 is a part of the primary antioxidant pathway that controls the expression of antioxidant proteins in glial cells [106]. According to recent research, brain injury may be lessened by activating the Sirt-1/NRF2 signalling pathway, which lowers oxidative stress and neuronal death [107]. It is known that one of the components selected for this combination—*Hericium erinaceus*—decreased aberrant APP overexpression and phosphorylated Tau levels [108]. Further, anthocyanin-enriched bilberry fractions were discovered to alter APP processing in a mouse model of Alzheimer’s disease [109]. Instead, *Centella asiatica* exerts its neuroprotective effects by regulating Sirt1 expression [110]. Lastly, PEA can restore cognitive function by decreasing the overexpression of pTAU protein [111] and restoring Nrf2 levels [112], thereby enhancing the compound’s potential as a neurodegenerative treatment. These data from the literature demonstrate the already established abilities of *Hericium erinaceus*, PEA, Bilberry extract, and *Centella asiatica* to counteract cognitive decline. Furthermore, these extracts are characterised by different mechanisms of action, leading to the hypothesis that the positive effects of the combination on a cognitive decline model may be due to the distinct beneficial effects induced by each agent.

This study showed how damage induction decreases the levels of the two proteins; in contrast, the new formulation under review increases and restores their levels, highlighting their protective activity at the brain level. Understanding the protective properties of the chemicals at the neuronal level also influenced the formulation choice.

These results suggest that a new formulation containing 400 µg/mL *Hericium erinaceus*, 0.2 µM PEA, 100 µg/mL Bilberry extract, and 250 µg/mL *Centella asiatica* may be beneficial in the treatment of cognitive decline. This formulation can cross biological membranes, including the intestinal barrier, and reach the intended location, exhibiting optimal results in the in vitro model of cognitive decline.

Even if this is an in vitro study and the combination’s long-term safety cannot be tested, different studies have already demonstrated the combination’s long-term safety of *Hericium erinaceus* [113], PEA [114], Bilberry extract [115], and *Centella Asiatica* [116], thus leading to the hypothesis that this combination can be used long-term without side effects.

Moreover, while the in vitro environment does not reproduce key physiological processes such as absorption, distribution, metabolism, and excretion, which are crucial in determining substance bioavailability in vivo, it nonetheless represents a well-established and widely accepted model for mechanistic studies. The concentrations used in our system are not intended to directly mirror clinical doses, but are chosen to elicit measurable and biologically relevant responses under controlled conditions. Although in vitro models cannot fully replicate the complexity of the human brain microenvironment, they provide a valuable and reproducible platform to investigate specific molecular and cellular pathways involved in cognitive decline. Our model enables a focused analysis of cell-level responses, free from the confounding variables present in in vivo systems, thus offering unique insights into potential mechanisms of action. While recognising these intrinsic limitations, our findings contribute meaningfully to the early-stage understanding of neuroprotective effects. Further validation in more physiologically integrated models will be needed to confirm the translational potential of the observed effects. In this context, the next step of this study could be developed to evaluate the effects of the combination of 400 µg/mL *Hericium erinaceus*, 0.2 µM PEA, 100 µg/mL Bilberry extract, and 250 µg/mL *Centella asiatica* in a clinical setting of patients affected by cognitive decline, considering the promising data obtained in vitro.

## 5. Conclusions

This in vitro investigation demonstrates that a novel combination of PEA, *Centella asiatica*, Bilberry extract, and *Hericium erinaceus* (named Eutrophic) can help alleviate oxidative stress without any cytotoxic effects, even at the intestinal level. This combination attenuates the harmful effects of several mechanisms related to neurodegenerative diseases, thus allowing the development of a new combination that can reduce the intracellular mechanisms underlying cognitive decline. Based on these findings, it is possible to hypothesise the use of a new oral nutraceutical supplement that can reduce every negative process linked to high levels of oxidative stress. However, data from more complex experimental settings are needed before introducing this combination into practice; specifically, data from an in vivo model or even better, from clinical trials are needed to ensure the effectiveness of this new combination even in more complex models.

## Figures and Tables

**Figure A1:**
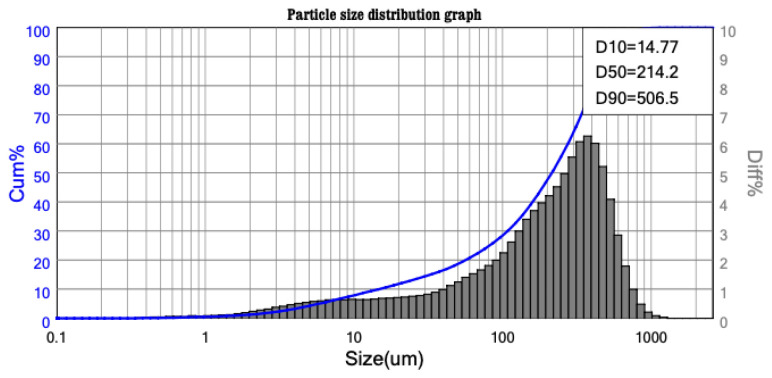
Particle size distribution analysis of PEA.

## Data Availability

The Laboratory of Physiology stores raw data to ensure permanent retention under a secure system. This study’s data are available from the corresponding author upon reasonable request.

## Abbreviations

The following abbreviations are used in this manuscript:

Adv DMEM-F12	Advanced Dulbecco’s modified Eagle’s medium/Nutrient F-12 Ham
APP	Amyoid precursor protein
ATCC	American Type Culture Collection
BBB	Blood–brain barrier
BDNF	Brain-derived neurotrophic factor
CNS	Central nervous system
DMEM	Dulbecco’s modified Eagle’s medium
ELISA	Enzyme-linked immunosorbent assay
FBS	Foetal bovine serum
GPx	Glutathione peroxidase
MDA	Malondialdehyde
NF-kB	Nuclear factor kappa-light-chain-enhancer of activated B cells
NGF	Nerve growth factor
NO	Nitric oxide
OEA	Oleylethanolamide
PEA	Palmitoylethanolamide
PNS	Peripheral nervous system
PPAR	Peroxisome proliferator-activated receptor
ROS	Reactive oxygen species
SIRT1	Sirtuin 1
SOD	Superoxide dismutase
TBARS	Thiobarbituric acid reactive substance
TEER	Trans-epithelial electrical resistance
TJ	Tight junction
TNFα	Tumour necrosis factor α
Trk	Tropomyosin receptor kinase family receptor

## Appendix A

**Table A1:** Summary table of the natural extracts used in the study.

Extract	Extract Solvent	Composition	% g/100 g of Content	Method	Origin
*Hericium erinaceus*	Water	Polysaccharides	58.52% of which:	ATR-IR	Fruit body
		80 mesh	14% of β−1,3-glucans		
PEA 60 mesh	Ethanol	Active ingredient	99.55%	HPLC	-
Bilberry extract	Organic Solvent (Ethanol, methanol and Acetone)	Anthocyanins	36.4% of which:	HPLC	Fruit body
			5.00% of Delphinidin		
			4.00% of Cyanidin		
			1.73% of Petunidin		
			0.74% of Peonidin		
		Free anthocyanidins	26.1%		
*Centella asiatica*	Ethanol and Water (25:1)	Total asiaticosides Madecassoside Dicaffeoylquinic Acid Free Flavonoids	11.59%	HPLC-MS	Herb
			4.66%		
			1.94%		
			1.68%		
